# The regulation of tumor‐suppressive microRNA, miR‐126, in chronic lymphocytic leukemia

**DOI:** 10.1002/cam4.996

**Published:** 2017-03-15

**Authors:** Daphne Guinn, Amy Lehman, Catherine Fabian, Lianbo Yu, Kami Maddocks, Leslie A. Andritsos, Jeffrey A. Jones, Joseph M. Flynn, Samantha M. Jaglowski, Jennifer A. Woyach, John C. Byrd, Amy J. Johnson

**Affiliations:** ^1^ Biomedical Sciences Graduate ProgramCollege of MedicineThe Ohio State UniversityColumbusOhio; ^2^ Division of HematologyDepartment of Internal Medicine and Comprehensive Cancer CenterThe Ohio State UniversityColumbusOhio; ^3^ Center for BiostatisticsThe Ohio State UniversityColumbusOhio; ^4^ Division of Medicinal ChemistryCollege of PharmacyThe Ohio State UniversityColumbusOhio

**Keywords:** CLL, ibrutinib, microRNAs

## Abstract

The introduction of miR profiling of chronic lymphocytic leukemia (CLL) patients with different cytogenetic profiles and responses to therapy has allowed incorporation of important miR‐mRNA interactions into the understanding of disease biology. In this study, we performed miR expression analysis using NanoString nCounter to discover differentially regulated miRs after therapy with the Bruton tyrosine kinase inhibitor ibrutinib. Of the differentially regulated miRs in the discovery set, miR‐29c and miR‐126 were confirmed using real‐time PCR to be upregulated in CLL patient cells with ibrutinib therapy. In the validation set, an inverse correlation was observed between miR‐126 levels and expression of its putative target p85*β*, an isoform of the phosphoinositide 3‐kinase p85 regulatory subunit. We found that mRNA for the host gene *EGFL7*, primary unprocessed miR‐126, and mature miR‐126 are all downregulated in CLL cells compared to normal B cells. Patients in later stages of disease have a greater decrease in miR‐126 expression compared to treatment‐naive patients, indicating that lower miR‐126 levels may associate with disease progression. Overexpression of miR‐126 in leukemia cell lines significantly downregulates p85*β* expression and decreases activation of prosurvival mitogen‐activated protein kinase (MAPK) signaling. These results implicate miR‐126 in the pathology of CLL.

## Introduction

MicroRNAs (miRs) are small noncoding RNAs that can guide RNA silencing 1. The importance of miR profiling has been well described in CLL, but target identification can be problematic because of the context‐dependent manner in which miRs function. In CLL, the downmodulation of miRs that target prosurvival proteins has been well studied. One of the early miR studies showed that miR‐15a and miR‐16‐1 were located within the commonly deleted chromosomal region of 13q14 in CLL 2, and follow‐up work showed these two miRs to be major negative regulators of the antiapoptotic protein B‐cell lymphoma 2 (BCL‐2) 3. It was also discovered that miR‐29 and miR‐181 could target the oncogenic prosurvival protein T‐cell leukemia/lymphoma protein 1 (TCL1) 4. More recently, associations between B‐cell receptor (BCR) stimulation and miR regulation have been reported, including a study showing that one of the most highly expressed miRs in CLL, miR‐150, could target GAB1 and FOXP1 5. As these two proteins enhance BCR signaling, this finding introduces the potential of miRs to regulate key intracellular pathways in tumor cells 5. Our group described the association of oncomiR‐155 with the BCR‐activated phenotype in CLL, its upregulation correlating with shorter patient survival, and its downmodulation following therapy with the BCR‐targeted agent ibrutinib 6.

In this study, high‐throughput miR expression profiling was performed to discover novel miRs that are modulated by ibrutinib therapy. Here, we show that miR‐126 is upregulated as soon as 1 month into ibrutinib treatment. *miR‐126* is located on chromosome 9 within the seventh intron of the epidermal growth factor‐like domain 7 (*EGFL7*) gene, and miR‐126 and EGFL7 expression patterns are typically similar within particular tissues 7, 8. Their expression is generally restricted to endothelial cells and hematopoietic progenitors, where these factors contribute to the development of the vascular system. Normally, miR‐126 expression is only maintained in highly vascularized tissues and can be reactivated in sites where angiogenesis is occurring as a part of normal or disease processes 7. The expression and role of miR‐126 in cancer development and progression has been previously interrogated in solid tumors cancers, where it was found to be downregulated in tumor versus normal tissue 8. miR‐126 is thought to be tumor suppressive through its ability to modulate angiogenesis, tumor growth, and metastasis 9.

In esophageal squamous cell carcinoma and colon cancer, miR‐126 levels are decreased and inversely correlate with that of p85*β*, a regulatory subunit of the phosphatidylinositol‐4,5‐bisphosphate 3‐kinase (PI3K) complex 10, 11. The PI3K complex is composed of a heterodimer of the p110 catalytic subunit and the p85 regulatory subunit that stabilizes p110 and controls its activation. PI3K, following activation via various receptor tyrosine kinases, phosphorylates PIP_2_ to PIP_3_ to allow recruitment of pleckstrin homology (PH) domain‐containing proteins to the membrane 12. This recruitment leads to activation of critical survival signaling pathways including PI3K, MAPK, NFAT, and NF*κ*B. A luciferase reporter assay showed that miR‐126 can bind the 3′UTR of p85*β* and reduce reporter activity, and introduction of miR‐126 into colon and esophageal cancer cell lines led to a decrease in p85*β* protein expression and downstream phosphorylation of protein kinase B (AKT) 10, 11. In support of these findings, miR‐126 overexpressing cells show reduced proliferation, migration, and colony formation 10.

miR‐126 has been shown in two studies to be downmodulated in CLL versus normal B cells 13, 14, and decreased miR‐126 expression was contributed to a gain in methylation at the predicted transcriptional start site in CLL cells compared to normal B cells 13. Despite these findings, little is known about the contribution of miR‐126 expression to CLL biology. Potential targets for miR‐126 have not been explored, and it has yet to be determined if miR‐126 plays a tumor‐suppressive role in CLL.

In our study, we perform miR expression analysis to elucidate the miR profile associated with ibrutinib therapy. We go on to interrogate the role of miR‐126 in CLL by evaluating the expression of miR‐126 and its host gene, *EGFL7*, in normal B cells as well as in CLL patients at different stages of disease. miR‐126 overexpressing cell lines were created to evaluate the interaction of miR‐126 and its target mRNA, *p85β*, as well as the phenotypic changes that induction of miR‐126 expression causes in leukemia cell lines. Our data indicate that miR‐126 expression negatively correlates with *p85β* in CLL patients and that miR‐126 can effectively target p85*β* in a cell‐line system.

## Materials and Methods

### Patient samples

Samples were collected from relapse or refractory CLL patients enrolled on OSU‐10053 (NCT01217749) and OSU‐11133 (NCT01589302) as previously reported 15, 16. Blood was obtained from patients with written informed consent in accordance with the Declaration of Helsinki and under a protocol approved by the Institutional Review Board (IRB) of The Ohio State University, and cryopreserved in freezing medium containing 10% dimethyl sulfoxide. Normal CD19^+^ B cells were obtained from anonymous donors as part of a second exemption protocol approved by The Ohio State University IRB. Samples were selected and processed as previously described 17.

### Reagents and antibodies

Anti‐AKT, anti‐phospho‐AKT (Ser^473^), anti‐phospho‐ERK1/2 (Thr^202^/Tyr^204^), and anti‐ERK1/2 antibodies were purchased from Cell Signaling Technologies (Danvers, MA). Anti‐p85*β* antibody was purchased from Abcam (Cambridge, MA). Anti‐GAPDH antibody was purchased from EMD Millipore (Darmstadt, Germany), and antiactin antibody was purchased from Santa Cruz Biotechnology (Santa Cruz, CA). Human anti‐IgM was obtained from Bethyl laboratories (Montgomery, TX).

### RNA extraction, Nanostring, and Quantitative RT‐PCR (qRT‐PCR)

RNA was extracted 6 and cDNA was prepared as previously described 6, 18. nCounter Human miRNA expression assay (version 1.3, NanoString Technologies, Seattle, WA) was performed to profile miR expression before and after 1 month of ibrutinib treatment. For miR studies, cDNA was prepared using the TaqMan^®^ MicroRNA Reverse Transcription kit (Applied Biosystems, Foster City, CA). Real‐Time PCR was performed using TaqMan^®^ Gene Expression Assays or TaqMan^®^ microRNA assays on the ABI ViiA7 Real time PCR system (Applied Biosystems).

### Cell transfection and maintenance

The acute lymphoblastic leukemia cell‐line 697 and the CLL cell‐line OSU‐CLL were confirmed to be mycoplasma free and were maintained as previously described 19, 20. The inducible Tet‐on transactivator (pRetroX‐Tet‐On; Clontech Laboratories/ Takara, Mountain View, CA) was stably introduced into the cell lines as previously described 21. The pri‐miR‐126 was introduced into the multiple cloning site of the retroviral construct, pRetroX‐Tight‐Puro (Clontech). The constructs, pRetroX‐Tight‐Puro (vector control) or pRetroX‐Tight‐Puro‐miR‐126, were used to stably infect the cell lines. Confirmation of the DNA sequence, production of viral particles, and infection of the cells were performed as described 21. Cells were selected with 1ug/mL puromycin and 250 *μ*g/mL G418. miR‐126 expression was induced with 1ug/mL of doxycycline (Clontech) for 48 h.

### Immunoblot analysis

Lysate preparation, protein quantification, and immunoblot analyses were performed as described 22, 23. Bands were quantified on an AlphaImager system (Proteinsimple, San Jose, CA).

### Statistical analysis

Differences in miR expression before and after ibrutinib treatment were estimated from linear mixed effects models to account for correlations among observations from the same patient. Models were fit using the ΔCT values to reduce skewness and stabilize variances. Pearson correlations among p85*β*, EGFL7, and miR‐126 were calculated. Analysis of the vector control versus the miR‐126 overexpression cell line was done by applying mixed effects models to the ΔCT values, and fold changes were estimated from the model. *P*‐values for the primary comparisons (miR‐126 vs. vector) were adjusted for multiple comparisons using Holm's procedure. Similarly, differences in signaling following BCR stimulation in the miR‐126‐expressing cell line compared to the vector control were performed using mixed effects models. An interaction test was used to determine if there were differences in the cell lines in stimulated and unstimulated conditions. Finally, expression levels of *EGFL7*, the full‐length precursor pri‐miR‐126, and mature miR‐126 were evaluated in normal B cells from healthy donors, treatment‐naive patients, and heavily pretreated patients using analysis of variance (ANOVA); p‐values were adjusted for multiple comparisons. Analyses were performed using SAS/STAT software, Version 9.4 (SAS Institute Inc., Cary, NC).

## Results

### Discovery and validation of miRs regulated by ibrutinib therapy

**Figure 1:**
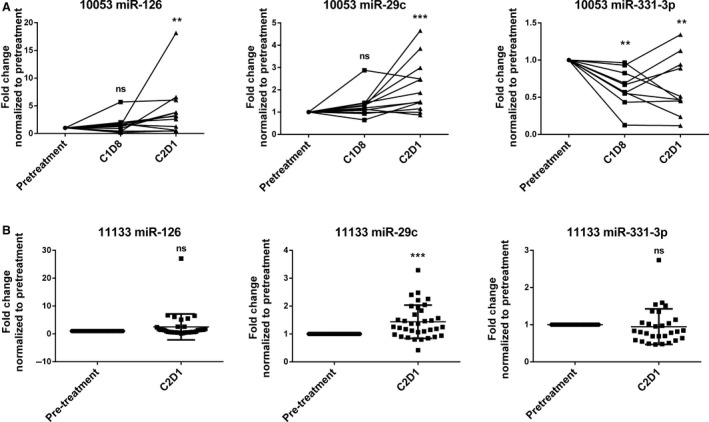
Validation of ibrutinib‐regulated miRs. (A) qRT‐PCR analysis of the miRs significantly regulated by ibrutinib in 12 patients treated on OSU‐10053. (B) qRT‐PCR of the OSU‐11133 validation set in 34 ibrutinib‐treated patients. ** *P* ≤ 0.01, *** *P* ≤ 0.001.

To determine if ibrutinib therapy can modulate miR expression in CLL patients, samples were obtained from eight patients at pretreatment, 8 days (C1D8), and 1 month (C2D1) after receiving ibrutinib therapy (420 mg/day) on clinical trial OSU‐10053 15. RNA from patient samples was pooled into two groups with four patients per group and assessed using the NanoString nCounter platform. Initial analysis of the output was done by eliminating low‐expressing miRs, and expression was normalized using an adjusted alpha level to include fold changes of at least 1.5 and allow for the possibility of five false positives. The list of potential miRs was narrowed down to include only miRs that changed significantly (*P* < 0.01) at C2D1 (Table 1). Of the significantly altered miRs, three were selected for validation by quantitative PCR based on their published functions in CLL biology or known associations in other cancer types 8, 24, 25. The results for miR‐126, miR‐29c, and miR‐331‐3p were validated using 12 patient samples—the original eight samples used in the NanoString analysis and four additional samples from patients treated on OSU‐10053 (Fig. 1A). miR‐126 was significantly upregulated at C2D1 (*P* = 0.009), but not at C1D8 (*P* = 0.700). Showing the same trend, miR‐29c was significantly upregulated at C2D1 (*P* < 0.001) but not at C1D8 (*P* = 0.140). miR‐331‐3p was downregulated at both C1D8 (*P* = 0.009) and C2D1 (*P* = 0.005). Following confirmation of the miR expression results, we validated these data using an independent sample set from a second clinical trial, OSU‐11133 16. Quantitative PCR was performed for the above miRs at the pretreatment and C2D1 time points in 34 additional patients (Fig. 1B). In this set, miR‐29c showed the same significant upregulation after 1 month of therapy (*P* < 0.001). miR‐331‐3p shows a trend toward downregulation, but the association did not reach significance (*P* = 0.083). Finally, miR‐126 expression was not significantly upregulated in the validation set (*P* = 0.219).

**Table 1:** miRs significantly regulated after 8 days and 1 month of ibrutinib therapy

	Fold Change Pre vs. C2D1	*P* Value
hsa‐miR‐155	−1.7252	0.0007
hsa‐miR‐532‐3p	−4.4134	0.0018
hsa‐miR‐331‐3p	−**1.9939**	**0.0023**
hsa‐miR‐29c	**1.6552**	**0.0044**
hsa‐miR‐2053	−2.6960	0.0053
hsa‐miR‐520e	1.9008	0.0091
hsa‐miR‐126	**2.0535**	**0.0094**
Cut off at *P*<0.01		

The microRNAs that are further interrogated are in bold.

We also evaluated if miR‐126 expression was associated with established prognostic markers. No significant associations were observed between miR‐126 expression and *IGHV* mutational status (*P* = 0.099) or cytogenetic abnormalities del13q14.3 (*P* = 0.635) or del17p13 (*P* = 0.635) (Table S1).

### miR‐126 levels show correlation with EGFL7 expression and p85*β* expression in CLL patients

We next studied published targets for miR‐29c and miR‐126 to determine if target or host gene modulation correlated with miR expression in CLL patients before and after ibrutinib therapy. The miR‐29 family has been previously reported to target the antiapoptotic protein MCL1 and the oncoprotein TCL1 3. In samples from OSU‐11133, MCL1 was downregulated after 1 month of ibrutinib therapy (*P* = 0.002, Fig. S1a), but its expression did not inversely correlate with miR‐29c expression (*P* = 0.474, Fig. S1b). TCL1 expression was not significantly downregulated after ibrutinib therapy (*P* = 0.481, Fig. S1c) and its expression was not correlated with miR‐29c expression (*P* = 0.449, Fig. S1d).

**Figure 2:**
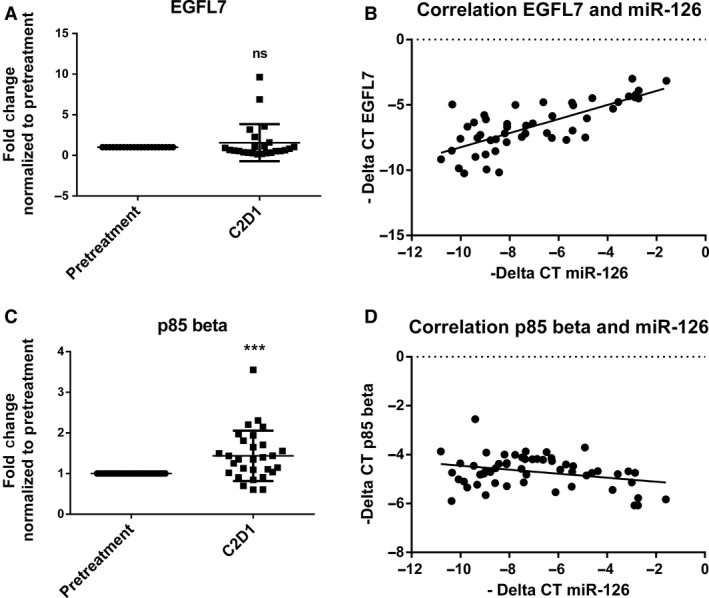
miR‐126 expression correlates with host gene, EGFL7, and target, p85*β*, expression in CLL patients. (A) qRT‐PCR analysis of miR‐126 host gene, EGFL7 mRNA expression evaluated in OSU‐11133 ibrutinib‐treated patients (*n* = 24). (B) Correlation plot using the –ΔCT of miR‐126 expression and *EGFL7* in patient before and after treatment with ibrutinib shows a positive correlation (*P* < 0.001). (C) qRT‐PCR analysis of miR‐126 target, p85*β* mRNA expression evaluated in OSU‐11133 ibrutinib‐treated patients (*n* = 29). (D) Correlation plot using the –ΔCT of miR‐126 expression and p85*β* in patient before and after treatment with ibrutinib shows a negative correlation (*P* = 0.021). *** *P* ≤ 0.001.

Because expression of miR‐126 has been reported to parallel that of its host gene *EGFL7* 13 *,* we interrogated *EGFL7* expression in CLL patient samples before and after ibrutinib therapy. *EGFL7* expression was not significantly modulated after treatment (*P* = 0.322, Fig. 2A). Although *EGLF7* expression was not regulated by ibrutinib, there was a strong positive correlation between the expression of miR‐126 and *EGFL7* in CLL patient samples before and after ibrutinib therapy (*P* < 0.001, Fig. 2B). These results indicate that miR‐126 is likely regulated through the same promoter as *EGFL7*. Previous reports show that miR‐126 can modulate PI3K signaling by targeting the regulatory subunit, p85*β* 10, 11. We therefore measured mRNA expression of p85*β* in both pre‐ and post‐ibrutinib CLL patient samples. Our results showed that p85*β* expression was indeed increased with ibrutinib therapy (*P* = 0.001, Fig. 2C), and that miR‐126 expression inversely correlated with p85*β* pre‐ and posttreatment (*P* = 0.021, Fig. 2D). Thus, miR‐126 has a positive correlation with its host gene, *EGFL7,* and an inverse correlation with its target, p85*β*, in CLL patients regardless of the change in expression postibrutinib therapy.

### miR‐126 expression decreases with CLL progression

**Figure 3:**
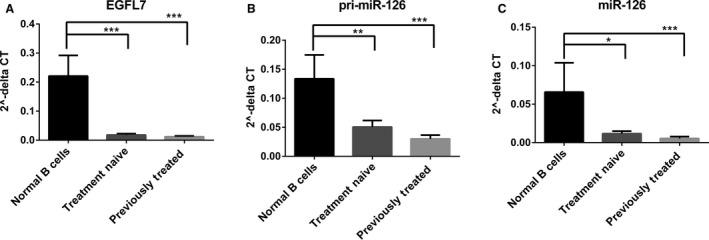
miR‐126 expression is decreased with CLL progression. (A) qRT‐PCR analysis of *EGFL7* in normal donor B cells (*n* = 6), treatment‐naive CLL patients (*n* = 14), and previously treated CLL patients (*n* = 15). (B) qRT‐PCR analysis of pri‐miR‐126 in normal donor B cells (*n* = 4), treatment‐naive CLL patients (*n* = 12), and previously treated CLL patients (*n* = 14). (C) qRT‐PCR analysis of mature miR‐126 in normal donor B cells (*n* = 6), treatment‐naive CLL patients (*n* = 15), and previously treated CLL patients (*n* = 15). * *P* ≤ 0.05, ** *P* ≤ 0.01, *** *P* ≤ 0.001.

Following our finding that miR‐126 expression correlates with *EGFL7* expression in CLL patients, we further studied the role of miR‐126 and its host gene in CLL progression. Expression levels of *EGFL7*, the full‐length precursor pri‐miR‐126, and mature miR‐126 were evaluated in normal B cells from healthy donors, treatment‐naive patients, and heavily pretreated patients (Fig. 3A–C). There was a similar trend in expression throughout miR processing from the host gene to the mature miR. After analysis by a one‐way ANOVA and adjusting for multiple comparisons, we found that treatment‐naive patients show decreased *EGFL7* (*P* < 0.001), pri‐miR‐126 (*P* = 0.009), and mature miR‐126 (*P* = 0.022) and in heavily previously treated patients (*P* < 0.001) compared to normal B cells. Comparing mature miR‐126 expression levels in treatment‐naive patients in early stages of disease versus in heavily previously treated patients in more advanced stages shows that miR‐126 expression decreases as leukemia progresses (*P* = 0.026).

### Overexpression of miR‐126 leads to decrease in p85*β* expression

**Figure 4:**
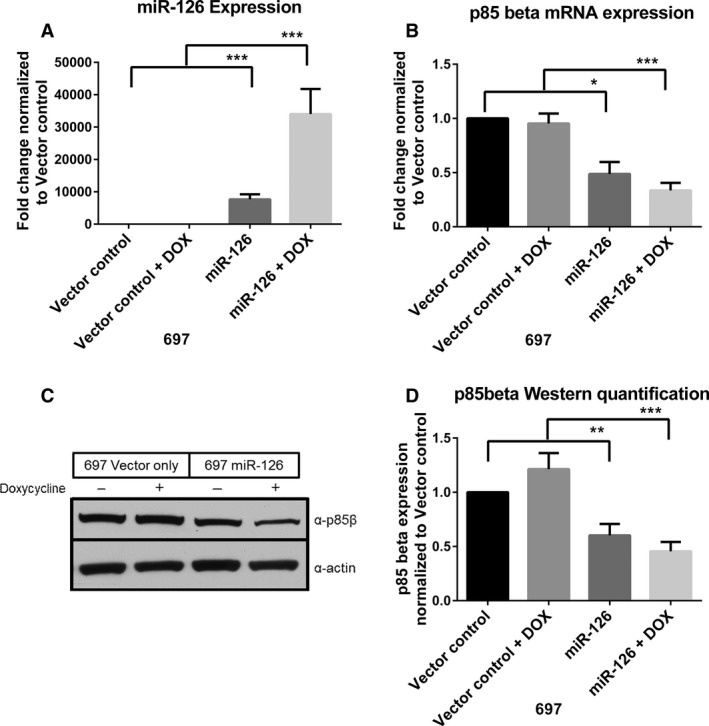
miR‐126 overexpression in B‐cell leukemia cells leads to downregulation of p85*β*. (A) qRT‐PCR analysis of miR‐126 expression in 697 cell lines infected with a control vector (VO) or a vector containing miR‐126 with or without 48 h induction with doxycycline (DOX) (*n* = 4). (B) qRT‐PCR analysis of p85*β* expression in the VO and miR‐126 cell lines with or without 48 h induction with DOX (*n* = 4). (C) Western blots showing p85*β* protein expression in the VO and miR‐126 cell lines with or without 48 h induction with DOX. (d) Protein quantification of the Western blot shown in c. p85*β* expression was normalized to actin and then normalized to the uninduced vector control cells (*n* = 5). * *P* ≤ 0.05, ** *P* ≤ 0.01, *** *P* ≤ 0.001.

To investigate how miR‐126 contributes to disease biology, miR‐126 overexpression cell lines were created using a retroviral vector system with a doxycycline‐inducible promoter to control miR expression. miR‐126 was successfully overexpressed in the 697 acute lymphoblastic leukemia cell line (*P* < 0.001, Fig. 4A) and the OSU‐CLL CLL cell line (*P* < 0.001, Fig. S2a) and could be further induced in both cell lines (*P* < 0.001). Target regulation was analyzed by quantitative PCR and immunoblotting. qRT‐PCR analysis in the 697 cells showed significant downmodulation of p85*β* mRNA after overexpression (*P* = 0.015) and doxycycline induction (*P* = 0.001) of miR‐126 (Fig. 4B). A similar trend was seen in the OSU‐CLL cell line before (*P* = 0.002) and after doxycycline induction (*P* < 0.001, Fig. S2b). Protein levels of p85*β* were also significantly decreased in the induced miR‐126‐containing cells versus the induced vector control (*P* < 0.001, Fig. 4C–D; *P* = 0.048, Fig. S2c‐d).

**Figure 5:**
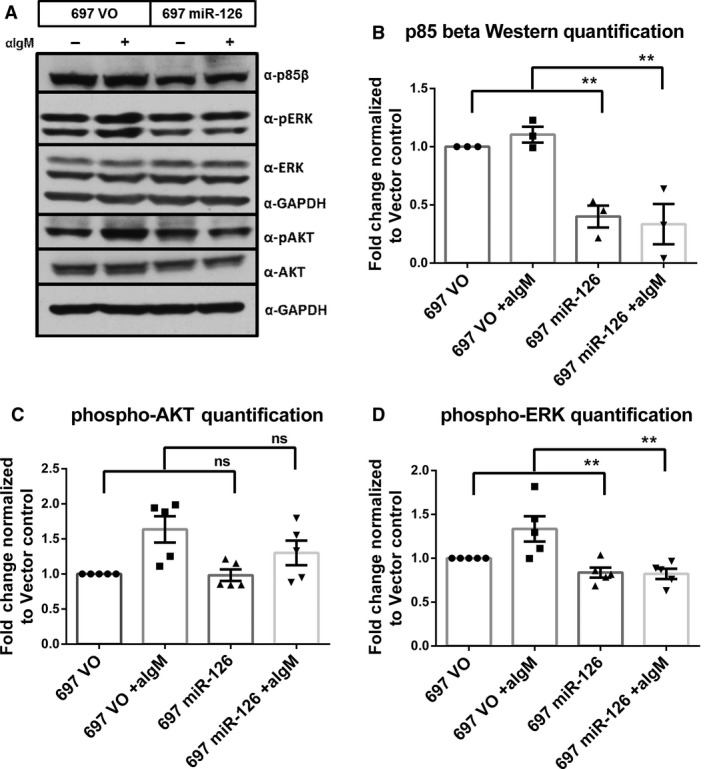
miR‐126 overexpression reduces prosurvival signaling after BCR stimulation (*n* = 5). (A) Western blot showing the vector control (VO) cell line compared to the miR‐126 overexpressing cell line with 48 h of DOX induction and before and after 15 min of plate***‐***bound anti‐IgM stimulation. (B) Protein quantitation analysis of p85*β* normalized to expression of GAPDH and then normalized to the expression of the unstimulated vector control cells. (C) Protein quantitation analysis of phospho‐AKT (pAKT) and AKT normalized to expression of GAPDH, next normalized by pAKT to AKT expression and fold change was determined by a final normalization to the unstimulated vector control cells. (D) Protein quantitation analysis of phospho‐ERK (pERK) and ERK normalized to expression of GAPDH, next normalized by pERK to ERK expression, and fold change was determined by a final normalization to the unstimulated vector control cells. ** *P* ≤ 0.01.

The necessity of p85*β* in the stabilization and activity of the PI3K complex led us to interrogate migration and proliferation in the miR‐126 overexpressing cell lines. We saw no change in proliferation or migration after induction of miR expression (data not shown). We next evaluated differences in prosurvival signaling following BCR stimulation with anti‐IgM antibody in the miR‐126 overexpressing cell lines compared to the vector control (Fig. 5). The miR‐126‐expressing cell line showed a significant decrease in p85*β* expression in both stimulated and unstimulated conditions (*P* = 0.002, Fig. 5A–B), although the differences in phosphorylation of AKT between the vector control cell line and the miR‐126 cell line were not significantly different (*P* = 0.478, Fig. 5C). However, in both IgM‐stimulated and unstimulated conditions, miR‐126 overexpressing cells showed a significant decrease in ERK phosphorylation compared to the vector control (*P* = 0.009, Fig. 5D), indicating a decrease in prosurvival signaling in miR‐126‐overexpressing cells.

## Discussion

The BTK inhibitor ibrutinib has provided extremely impressive clinical benefit in the majority of CLL patients treated to date. To our knowledge, this report provides the first description of high‐throughput microRNA profiling in peripheral blood CLL cells of patients after ibrutinib therapy. Following our initial analysis showing differential regulation of miRs as soon as 8 days after initiation of ibrutinib treatment, we validated expression of three miRs, miR‐29c, miR‐331‐3p, and miR‐126. miR‐29c was previously published to be differentially expressed in CLL versus normal B cells 3, 4. It was also found to be downregulated in patients with an abnormality in TP53, which is associated with poor prognosis in CLL 24. Here, we show that miR‐29c is upregulated with ibrutinib treatment in both our discovery and validation set. miR‐331‐3p has been found to be important in prostate and gastric cancer by targeting aberrant signaling pathways 25, 26. Although miR‐331‐3p expression was significantly downregulated in the discovery data set, we were unable to confirm its modulation in our validation set. miR‐126 is known to be downregulated in CLL versus normal B cells, likely due to increased transcriptional start site methylation in CLL 13, 14. miR‐126 was confirmed to be upregulated with ibrutinib treatment in the discovery dataset, but this increase was not significant in a larger group of patients enrolled on OSU‐11133. Despite this, we found that miR‐126 expression positively correlated with expression of its host gene *EGFL7*, and inversely correlated with its putative target p85*β*, in CLL patients both before and during ibrutinib treatment.

The importance of miR‐126 has not been further interrogated in CLL, but its role as a tumor suppressor through its ability to target PI3K signaling has been studied in solid tumors 8, 10, 11. Here, we show that not only is miR‐126 downregulated in CLL, but its expression is lower in previously treated patients compared to untreated patients. This finding suggests that miR‐126 is downmodulated as leukemia progresses. With the additional finding that miR‐126 expression is inversely correlated with the expression of p85*β* in CLL, we studied miR‐126 function by creating miR‐126‐overexpressing B‐cell lines. In these cells, we observed significant downregulation of p85*β* at both the transcript and protein levels, indicating transcriptional degradation as the likely targeting mechanism. The introduction of the microRNA could also be modulating unknown targets that could have an impact on p85*β* expression. It has been shown in other disease states that miR‐126 could modulate VEGF 27 and IRS‐1 28. These proteins can modulate downstream PI3K signaling 27, 28. Other relevant upstream signaling within these cells could be impacting p85*β* regulation.

We next investigated if miR‐126 overexpression and concomitant loss of p85*β* would lead to differences in prosurvival signaling following BCR stimulation. It was previously published that phosphorylation of AKT, which is downstream of PI3K activation, could be decreased after introduction of miR‐126 10, 11. We show here that cells show a slight increase in AKT phosphorylation upon BCR stimulation, but there is not a difference in phosphorylation between the vector control and miR‐126‐overexpressing cells. However, the phosphorylation of ERK is decreased in the miR‐126‐overexpressing cell line, indicating that the presence of this miR indeed can reduce prosurvival signaling in these cells. It was previously shown that miR‐126 can target both PI3K and ERK signaling through multiple targets, while performing its role in angiogenesis and vascular integrity 29. Thus, it is likely that miR‐126 affects other unidentified targets that can also impact signaling. Also, p85*β* might not the dominant regulatory isoform critical for PI3K signaling in these cells, but has an alternative function. The function of p85*β* in lymphocytes has not been extensively studied compared to p85*α*. Phosphatidylinositol 3‐kinase regulatory subunit alpha (*PIK3R1)* is known to encode p85*α*, p55*α,* and p50*α*, whereas phosphatidylinositol 3‐kinase regulatory subunit beta (*PIK3R2)* produces p85*β* 30. Both p85*α* and p85*β* are considered to be ubiquitously expressed and are known for their function in stabilizing p110 to regulate PI3K complex activity. These regulatory proteins have also been indicated in other functions independent of the PI3K complex 31. P85*β* is known to be mutated and overexpressed in some cancers and may play a role in tumor progression and invasiveness 32, 33, 34. It can complex with focal adhesion kinase (FAK), allowing for enhanced stability and maintenance of cell adhesion. In metastatic melanoma, the interaction among FAK, p85*β,* and Cdc42/Rac leads to enhanced actin polymerization and formation of invadopodia 33. p85*β* was also reported to play a p110‐independent role in the unfolded protein response (UPR) in endoplasmic reticulum (ER) stress. p85 could associate with X box‐binding protein 1 (XBP‐1), a critical protein in orchestrating UPR, and increase its nuclear translocation during insulin signaling 35. A p110‐independent function for p85*β* associated with BCR signaling has yet to be described.

miR profiling in CLL has had an impact on understanding disease biology and provides useful biomarkers. The pivotal identification that miR‐15a and miR‐16‐1 are located within the minimally deleted region of the most common genetic aberration in CLL, del13q14, led to a flurry of studies identifying miRs that were differentially regulated in CLL patients 2. Current studies are focused on determining how miRs contribute to the disease phenotype. The expression of specific miRs and their interactions with putative targets can provide key information regarding cell behavior and pathology. This study presents a new and unique profile of miRs modulated with ibrutinib therapy that allowed for the identification of miR‐126 as a potential factor in CLL. We confirm that miR‐126 is downmodulated in CLL, and that one of its roles is related to its modulation of the PI3K regulatory subunit, p85*β*.

## Conflict of Interest

The authors have nothing to disclose.

## Supporting information


**Table SI.** Patient characteristics and miR‐126 expression
**Figure S1.** *MCL‐1* expression is decreased with ibrutinib therapy, but does not correlate with *miR‐29c expression*.
**Figure S2.** miR‐126 overexpression in the OSU‐CLL cell line decreases p85*β* expression.
