# Supplementary material for: The regulation of tumor‐suppressive microRNA, miR‐126, in chronic lymphocytic leukemia
Source: Cancer Med. 2017 Mar 15;6(4):778–87. doi: 10.1002/cam4.996 (PMC5387133; doi:10.1002/cam4.996)
Supplement: Supplementary file 1 — Table SI. Patient characteristics and miR‐126 expression Figure S1. MCL‐1 expression is decreased with ibrutinib therapy, but does not correlate with miR‐29c expression. Figure S2. miR‐126 overexpression in the OSU‐CLL cell line decreases p85β expression. [file CAM4-6-778-s001.docx]

**Supplemental Table I. Patient characteristics and miR-126 expression**

| **Outcome** | **Cytogenetics** | **Patient Number** | **Estimated Fold Change** | **95% CI** | **p-value** |
| --- | --- | --- | --- | --- | --- |
| miR-126 expression | 13q14.3: yes vs. no | 16, yes; 16, no | 0.71 | (0.2, 2.51) | 0.635 |
|  | 17p13.1: yes vs. no | 13, yes; 19, no | 0.74 | (0.2, 2.67) | 0.635 |
|  | Unmutated vs. mutated | 25, UM; 8, M | 4.38 | (1.14,16.82) | 0.099 |

**Supplemental figure 1. *MCL-1* expression is decreased with ibrutinib therapy, but does not correlate with *miR-29c expression.*** (a) qRT-PCR analysis of miR-29c target, MCL-1 mRNA expression evaluated in OSU-11133 ibrutinib treated patients (n=24). (b) Correlation plot using the –ΔCT of miR-29c expression and *MCL1* in patient before and after treatment with ibrutinib shows no significant correlation. (c) qRT-PCR analysis of miR-29c target, *TCL-1* mRNA expression evaluated in OSU-11133 ibrutinib treated patients (n=29). (d) Correlation plot using the –ΔCT of miR-29c expression and *TCL-1* in patient before and after treatment with ibrutinib shows no significant correlation. * p≤ 0.05, ** p≤0.01, *** p≤ 0.001.

**

**

**Supplemental Figure 2. miR-126 overexpression in the OSU-CLL cell line decreases p85β expression.** (a) qRT-PCR analysis of miR-126 expression in OSU-CLL cell lines infected with a control vector (VO) or a vector containing miR-126 with or without 48 hour induction with doxycycline (DOX) (n=6). (b) qRT-PCR analysis of *p85β* expression in the VO and miR-126 cell lines with or without 48 hour induction with DOX (n=6). (c) Western blots showing p85β protein expression in the VO and miR-126 cell lines with or without 48 hour induction with DOX. (d) Protein quantification of the western blot shown in c. p85β expression was normalized to actin and then normalized to the un-induced vector control cells (n=6). * p≤ 0.05, ** p≤0.01, *** p≤ 0.001.

**

**
